# Whole heart coronary MRA at 3.0T: a comparison between multi and single- RF transmission

**DOI:** 10.1186/1532-429X-13-S1-P233

**Published:** 2011-02-02

**Authors:** Masaki Ishida, Amedeo Chiribiri, Shazia Hussain, Andrea J Wiethoff, Geraint Morton, Matthias Paul, Rene Botnar, Eike Nagel

**Affiliations:** 1 King's College London Division of Imaging Sciences and Biomedical Engineering, London, UK

## Introduction

On high field MRI scanners uniform radio frequency (RF) excitation over the entire field-of-view is often challenging with single-channel RF transmit coils. This may cause a reduction of image quality (e.g. shadowing artifacts). This problem is most pronounced in sequences which heavily rely on a homogenous magnetic field, such as steady state free precession (SSFP) sequences, despite having been shown to yield best results for MR coronary angiography at 1.5T. Multi-channel RF transmission (Tx) has been shown to significantly improve the RF (B1 field) uniformity in high-field MRI. More accurate knowledge of the local B1 field also allows to better predicting the local specific absorption rate (SAR), thereby allowing for shorter repetition times (TR). Altogether, this may allow for the use of SSFP sequences at 3T and thus to combine the high CNR benefits of 3D SSFP with the high SNR advantages of 3T for whole heart coronary MR angiography (WHCMRA).

## Purpose

The purpose of this study was to evaluate if the use of a multi-channel RF transmit coil can improve image quality of WHCMRA at 3.0T compared to a conventional single-channel RF transmit coil.

## Methods

Ten healthy volunteers were scanned (Achieva, 3T, 32-channel coil, Philips Healthcare, Best Netherlands). After preparatory scans for localization of the heart, B1 calibration and identification of the coronary resting period, 3D transverse WHCMRA images were acquired using a fast segmented gradient echo (TFE) and a SSFP sequence with T2 preparation and fat saturation (SENSE factor = 2; full Fourier encoding; acquisition duration per cardiac cycle <120ms; navigator gating window = 5mm; resolution = 1.5x1.5x1.5mm, k-space encoding = Cartesian). The following 3 settings were compared: TFE, SSFP, SSFP+TX (TR/TE/flip = 4.0/1.16ms/20o, 5.0/2.5ms/70o, 4.5/2.3ms/70o, respectively). The acquisition order was randomized in each subject. Objective values such as SNR, CNR and vessel sharpness were determined for the right and left coronary artery systems. Two blinded, expert reviewers assessed a subjective quality score.

## Results

**Figure 1:**
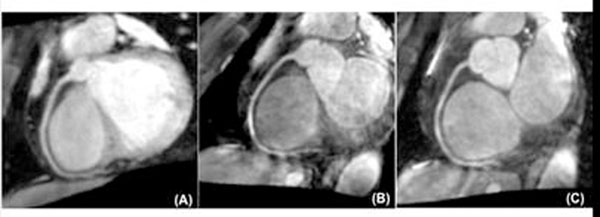
Right coronary scan with TFE (A), SSFP (B) and SSFP+Tx (C)

Mean actual scan time was between 9-11 minutes. Objective and subjective image quality for TFE and SSFP+Tx was comparable and substantially higher than for SSFP (Table 1, 2; Representative image, Figure 1). Banding artifacts were observed in all SSFP sequences.

**Table 1:** Objective evaluation of WHCMRA image quality.

		TFE	SSFP	SSFP+Tx	One-way ANOVA	Post test
Vessel sharpness (%)	RCA	35.2±8.6	37.7±7.2	32.6±5.5	p=0.308	-

	LAD	40.5±4.7	37.8±5.6	41.6±6.2	p=0.294	-

	LCx	32.4±5.5§	40.2±7.5§	38.4±6.1	p=0.030	§p=0.031

SNR muscle	RCA	13.2±3.7	9.5±3.4	12.8±4.7	p=0.099	-

	LCA	11.0±2.7§	7.4±2.8§¶	11.8±3.4¶	p=0.007	§p=0.034, ¶p=0.008

SNR blood	RCA	19.4±4.2	16.3±6.5	21.6±7.0	p=0.154	-

	LCA	16.9±3.2	13.1±4.8§	22.0±6.0§	p=0.001	§p=0.001

CNR	RCA	6.2±1.6	6.8±3.5	8.9±4.3	p=0.180	-

	LCA	6.0±13§	5.6±2.5¶	10.2±4.2¶§	p=0.002	§p=0.009, ¶p=0.005

**Table 2:** Subjective evaluation of WHCMRA image quality

		TFE	SSFP	SSFP+Tx	Kruskal-Wallis Test	Post test
Image qualituy score	RCA	2.9±0.6	1.9±0.7	2.6±0.9	p=0.22	-

	LAD	2.0±0.5	2.2±0.9	2.9±0.9	p=0.55	-

	LCx	2.6±0.8	2.5±0.5	3.0±0.7	p=0.201	-

1: poor (coronary vessel barely evident or noisy image) 2: moderate (coronary vessel visible but diagnostic confidence low) 3: good (coronary artery adequately visualized and diagnostic quality image) 4: excellent (coronary artery clearly depicted)

## Conclusion

SSFP WHCMRA benefits from multi-transmit RF transmission. This may be explained by the added benefit from an improved SAR model (shorter TR) and improved B1 homogeneity. However, banding artifacts still affect SSFP WHCMRA image quality when compared to the conventional TFE sequence.

